# Proceedings of the Maryland and District of Columbia Dental Society

**Published:** 1879-10

**Authors:** 


AMERICAN JOURNAL

OF

DENTAL SCIENCE.

Vol. XIII. THIRD SERIES—OCTOBER, 1879. No. 6.

### ARTICLE I.

*Proceedings of Maryland and District of Columbia Dental Society.—Annual Meeting.*

BALTIMORE, Tuesday Sept. 2nd, 1879.

The meeting was called to order by the President, Dr. J. C. Smithe, at 11 A. M. Prayer was offered by the Rev. Dr. Perry. The Secretary called the roll and reported a quorum present. Dr. T. S. Waters, of Baltimore, delivered the following address of welcome:

*Mr. President and Gentlemen of the State of Maryland and the District of Columbia Dental Association:*

In the performance of the very pleasant duty that has been assigned me I am here this morning to speak words of welcome to you as members of an earnest and honored profession, assembled to promote its advancement. While I feel that it is my province as a member of the dental profession and this Association, to work rather than indulge in attempted oratory; still my heart goes out to you and would have my unpracticed tongue utter the language of good-will and hearty welcome. In the name of the dental profession; in the name of the three hundred and fifty thousand citizens of this great city, and within the walls of the oldest Dental College in the world, I bid you a hearty welcome.

Drinking deep draughts of inspiration from the memory of the past life and works of one of her most self-sacrificing and earnest working citizens, I would remind you that Baltimore, ever rich in great and good men, gave to the world its first Dental College. And by the efforts of the illustrious Chapin A. Harris and his worthy compeers, erected an enduring monument, better than marble or bronze, that shall serve to keep their memory green as long as our profession shall live.

The great good this institution has done, and is still doing, not only in our country, but throughout the entire world, can never be told. Since eighteen hundred and thirty nine, when it began its course of instruction, to the present time, its influence and example have been ever operating to secure a high order of education, and instil into its graduates a high sense of the dignity and honor of our profession. For five years she battled alone against prejudices and selfish ignorance, when she was joined by her sister institution of Ohio, and to-day no less than fifteen Faculties are following in her illustrious wake of labor and usefulness.

When Dentistry, first struggling into life, cried out for recognition from existing professions, Medicine, her big sister (or as some would have it her mother) in her lofty pride, disdained to extend to her youngling a helping hand, or to lend a sympathising voice; but as the red and blue of the illiterate barber's pole no longer mark the abode of the practitioner of surgery, (the now petted foster-child of the science of Galen and Hippocrates) so too, our profession has by its industrious and ambitious devotees, been elevated from the barber's stool and old-fashioned turnkey, to the elegant cushions of the latest improved dentist's chair, and improved instruments; and to-day threatens to invade the most sacred precints of the elder professions, and cull the rich fragrant flowers of popular favor so jealously guarded in the past. But yesterday despised, to-day the acknowledged and courted rival, bestowing upon suffering humanity not health alone, but restoring beauty itself, and discarding the former dread of the operation for the now blissful peace of a dreamy sleep. To-day, Medicine, in thanksgiving for the gift the genius of a Wells has bestowed Anæsthesia cries out, "Hail sister! God speed in your good work of helping us to make life less of burden in the many ills sin has made flesh heir to."

Born amid the snows of a relentless winter, alone the honored profession of our choice was compelled to weather the rough, bleak storms; alone she withstood the drenching showers of early spring, till now we are scenting the sweet blossoms of a sunny May, affording abundant promises of the rich harvest with which the Autumn will reward us. But let us not forget that as the price of liberty is eternal vigilance, so the price of success is nev r-failing effort. While our past has been rich in blessings, we should remember that these blessings came by the permission of a kind Providence, as the result of the constant, fearless exertions of those who have ever labored earnestly for the advancement of our profession, *forgetting self* in their noble toil. To-day we stand in the places of the honored dead, and if their cold lips could speak to us, they would call upon us to go on in the way *their* feet would have trod, until we, like them, weary and worn, shall have strength to go no further. The future of our profession will depend upon the honorable bearing and the diligent efforts for its promotion, of those who have devoted themselves to its work. Individual effort and labor, can accomplish a great deal, but it is to Associations that we must look for the greatest good and success. It was a recognition of this truth which a few years ago gave rise to the organization of this Association. Since its organization, we have seen the promises of its originators *more* than fulfilled, and in looking back we find much of congratulation for the past, and of hope for the future.

Noticeable among the events of the year has been the union of the oldest Dental College with the youngest, blending the reputation of age with the vigor and originality of youth, the better and more successfully to do battle in the good and worthy cause of dental education. The mantle of the illustrious Harris has fallen upon the shoulders of one who has ever been zealous for the good of the profession of his choice, ever laboring earnestly for its advancement, and for a higher and more elevated standard of excellence.

This Association is now working successfully under a division of labor, (that of sections) rendering that possible to the specialist, which to the dentist (devoted however faithfully and laboriously to the study and practice of dentistry as an entire science and art) was impossible, for which it is indebted to the fertile brain of a R. B. Winder; nor should we forget his very able and comprehensive scheme of organizing a grand National Association, which knows no North, no South, no East, no West, but organized to accomplish the greatest good to the greatest number, and which was so forcibly presented to the American Dental Convention at its last meeting at Saratoga. The organization of this grand National body will certainly be effected at a called meeting of the National bodies now in existence, to be held in New York in September, 1880. In view of this fact, I desire to call the attention of this Association to one of the many very beautiful features of this National Organization,—it is that of membership. In order to become a member of and representative in this body, it is requisite that you should be a member of a State Dental Association, thereby guarding its rolls against any unprofessional *uncleanness*, and placing the responsibility (if any) upon the shoulders of the Association from which such a representative comes. In view of this feature, gentlemen, let us see to it that our rolls are kept clean and undefiled. Let us adopt such rules and measures as shall exclude from membership any one who would reflect discredit upon our Association. Let us cultivate a higher professional standard, and show to the public a true, honest and professional character, and thereby become bright luminaries in this Grand National Association.

And now, gentlemen, I bid you welcome to the good work you have here to perform in this grand old Monumental City, and in behalf of my professional brethren, I not only welcome you to her public squares and beautiful parks, but to places dearest far than all others, our houses and our homes.

The President then delivered the annual address, as follows:

*Gentlemen of the Maryland and District of Columbia Dental Association:*

It has been your pleasure to honor me with the position of presiding officer, at this our annual meeting.

I cannot express too earnestly the grateful feelings I have towards you for this mark of esteem. And while I feel that some among you would fill the position more suitably, on account of their years of experience and important services rendered to the profession, yet I assure you none could feel more proud, and none could enter upon the discharge of the duties involved with greater zeal, or with a keener sense of their responsibility. I pray you, gentlemen, to aid me with your counsel, to view my shortcomings with charity, and to help my inexperience with your kindly counsel and advice.

We once more hold our deliberations in this distinguished city, the scene of many meetings, to again experience those kindly greetings and attentions which Baltimoreans know so well how to offer. And it gladdens my heart to meet you once more in the parent city of Dental education, where those have lived and labored for the advancement of our profession whom we delight to honor, and where now prosperously exists the oldest Dental College in the world: a prestige that time cannot efface. While the advancement of our noble profession is so thoroughly illustrated by our numerous publications, it would be a needless waste of time for me to recapitulate the changes and the progress that has taken place during the past years. That will be developed as we proceed with our discussions.

In education, there have been changes here that we all hope and trust will be of great benefit in this, that we have one College in place of two. This change, no doubt, will favor a higher standard of education, and will benefit the profession at large, as well as the present institution. This example, I hope, will be followed by other cities, consolidating all into one, making that the college par excellence; selecting the best talent for instruction; infusing vigorous blood into the surviving institution, to give it added impetus as well as a wider field of usefulness.

To quote from an address of Prof. Hitchcock: "Our educational system I feel constrained to say, is, in my judgement, seriously defective. I am honestly of the opinion that our American civilization is over-doing itself professionally, not in quality, but in quantity. I know too, very well, that no profession is crowded near the top, but too much crowding at the bottom hurts the top. Under present conditions, the next best thing to be done is to inaugurate everywhere a system of rigid examinations. Such examinations may thin our ranks for a time, perhaps permanently. If only for a time, the wisdom of the policy will soon be vindicated. If permanently, it will prove that our ranks should have been thinned long ago."

This, in my opinion, is applicable to our profession now, and fully expresses my views on this vital point—our education.

I sincerely trust that in our discussions we shall be courteous and charitable to all. The points of view from which any given subject is considered by a public assemblage like the present, are, necessarily, nearly as many as the numbers of those considering it, and as varied as the changeful colors of the chameleon. The solecism that "Orthodoxy is my doxy, and heterodoxy is every body else's doxy," should find no place and no acceptance here.

We do not all bring to a subject the same lights of training or experience, any more than we are all equally rapid as thinkers or as reasoners, while nature has not gifted all of us with the same command of language. Many who are not fluent talkers, become, perforce the closest listeners, reasoning frequently more closely than if they were included in the ranks of ready speakers. To those, therefore, whom modesty or the fear of committing errors of diction, debars from taking a more prominent part in our discussions, let us be courteous and kindly in our intercourse, remembering that the possibility of error is common to us all, and that those who may possibly be silenced by ridicule are not convinced or won to change of views, for ridicule is not argument any more than abuse is reason.

I regret that the impoverished condition of our treasury is such, that I feel it my duty to call the attention of the Association to it. We are in debt to our Treasurer and to the printer heavily. This would not be the case if all dues were regularly paid. I would suggest that we discontinue printing the proceedings in future.

I feel diffident in speaking to you about the seeming lack of interest on the part of many for our Association. There are many that do not attend, or if so, but for an hour or two daily. A faithful few are always here, and they have born the burdens. Let us look to it that each shall bear his proper part. The Scripture doctrine "Bear ye one another's burdens," is not applicable to us, at least does not seem to be successful with Dental Associations.

In conclusion, I earnestly request your assistance to promote the most perfect harmony, as well as to aid me in expediting as rapidly and thoroughly as may be the business of this Association.

Gentlemen, for your kind attention, I thank you.

On motion of Dr. H. B. Noble, the reading of the minutes was dispensed with, as they were printed and in the hands of the members.

Election of members being in order, Dr. C. E. Duck proposed the name of Dr. A. J. Volck, of Baltimore, for active membership in the Association. Dr. Volck was duly elected a member.

Dr. Winder proposed the names of Prof. F. J. S. Gorgas, Dr. John C. Uhler, Dr. Albert Price and Dr. Wm. A. Mills for active membership, all of whom were duly elected.

Dr. Winder proposed the names of Drs. Martin B. Scott, I. E. Atkinson and Prof. Thomas E. Latimer, as honorary members of the Association. All were declared honorary members.

Under reports of business committees, Dr. Winder, chairman of executive committee, recommended that the hours for Clinics be from nine to eleven A. M.; to go into session at eleven and continue until four. Evening sessions at eight P. M. and continue until adjournment.

On motion of Dr. E. P. Keech, Dr. Winder's report was adopted.

Reading and discussion of papers being next in order, the President called them in order. Sections one, two, and three were held until the following day, when more papers would be ready.

Dr. J. J. Caldwell, of Baltimore, Chairman of Section four, introduced Dr. Martin B. Scott, of that city, who read the following paper on

### Syphilis of the Teeth, Hair, Nails and Skin.

To discuss pathological lesions it is necessary to know the physiological functions of the tissues are involved. Physiology is the basis of pathology. We must study the conditions of health in order to understand the alteration in function caused by disease. Were I addressing an assemblage of physicians alone, I should have no hesitation in plunging into the anatomy and physiology of the teeth, because I would be aware that very few doctors have given themselves the trouble to inform themselves upon this subject, being content to remand all cases involving these organs to their brothers who have made the oral cavity a specialty. But I feel in this presence, much as I once heard a distinguished lawyer and advocate say of his own feelings when arguing a chancery cause before Chief Justice Taney ; that he could not divest himself of the fact that the Chief Justice knew a vast deal more of the subject than he did. Such is a source of serious embarrassment to me on this occasion. I feel that I am in the presence of the masters of this branch of the profession. But at the same time I am reassured by the consciousness that you will look kindly on me in my effort to do justice to the subject.

I was much gratified when consulting some of your standard works, to find that gentlemen of your profession differed somewhat after the same fashion as doctors differ. As I have said, somewhere, what would become of the profession if doctorsknew it *all*, and there was a dead level of equality. What would be the condition of this very respectable planet of ours, if medical schools did not annually turn out hundreds of unfledged, half taught Doctors, to slay their thousands before they learn how to treat disease, I leave you to imagine ; or if all diseases could be once more shut up in Pandora's box ; or if misery still kept death tied up in the pear tree, there would be an end of your branch of the profession ; there would be an end of mine, unless we should then turn our attention to discover death, and set him to work.

In no countries is death so much feared and regarded with such horror as in Christian lands, which arises, I suppose, from the fact that they give us an account of a hell as well as a heaven. But in truth, death is the only friend that man cannot be deprived of, and for my part, should I ever discover the elixir of life, in my regard for the profession I shall keep it a profound secret. So long life to death!

But I find that there is one subject on which all seem to agree, viz, that *teeth* belong to the class of dermoid tissues, and therefore, have you deemed it fit to select a physician to appear before this Association to consider the effects which Syphilis impresses upon the teeth, the skin, hair, and nails.

For myself, I consider the classification of the teeth as dermoid tissues as very strained. The celebrated anatomist, Cruveilier, says the teeth pertain to the epidermic system, organs analogous to the nails and hair, because they present a lamellated texture like nails and hair, and are analogous in their mode of development to the horns, nails and hair. That like these, they are wanting in the phenomena of nutrition; are formed layer by layer and are not liable to a renewal of the substances which compose them. Products of transudation—inorganic bodies.

This account will scarce pass current with this Association if Salter and Tomes are recognized authorities. But strange as it may appear, although the above description is not true in any particular, yet the authorities above mentioned arrive at the same conclusion, and I believe that the opinion universally obtains among you that the teeth must be classed as dermoid tissue. Salter seems to base his opinion upon the belief that the teeth are developed from the integuments of the mouth. Tomes tells us that the enamel is a special development from the epithelium of the mouth —dentine from the deep-lying parts of the mucous-membrane. Or again, he says that the dentine germs and consequently the dentine are indisputably derived from the *connective* tissue of the mucous-membrane immediately subjacent to the epithelium. The enamel organs are modified epithelium of that same mucous-membrane. Cruveilier tells us that the teeth do not inflame, are never the seat of tumours, or any pathological production. Salter tells us of necrosis, exostosis, of abscess, of warty teeth, and Broca has published a treatise on tumours of the teeth.

The teeth were long considered as true bone, but the differences do not admit of this classification. I make bold to announce the opinion that neither classification is correct, whether we consider the teeth histologically, or from a physiological point of view. The functions of the teeth are varied, and differ in every respect from the skin and epidermis. They occupy the sweep of the upper and lower maxillæ, serve to preserve the symmetry of those organs, so that their growth may proceed to completion to serve the purposes intended by nature.

The teeth are necessary to clear articulation—a part of our telephonic arrangement; and lastly they are the organs for mastication. We hence, in view of their functions, find a highly organized and complex arrangement. Each tooth is a separate organ; it is implanted in a depression in the jaw called a socket; it is retained in its place by a peculiar arrangement. The socket is lined by a periosteal membrane, improperly called a root membrane, which is richly supplied with nerves and blood-vessels; it is continuous with the gums which are moulded over the teeth. The teeth themselves are constituted of a nervous and vascular pulp, surrounded by an ivory case over the crown of which is laid the dense enamel, covered by a membrane which, in order to carry out the analogy to the skin, has been named enamel cuticle. The fang and neck being covered by an envelope which in structure resembles bone, called *crusta petrosa*, or cementum.

Naturalists classify objects in nature from certain resemblances and differences observed between them. Thus, man has been classed by the advanced scientists with the ape, both have the perineus longus muscle which proves that the hand of the ape is a foot—(Huxley.) They class certain substances as muscles from similarity of tissue and function. In all correct classification, function is the basis, because function is the result of structure. No two substances of the same structure and tissue perform different offices in the animal economy. Cruveillier relying upon similarity of structure, classes teeth with the epidermic system; but the only lamellated texture about the teeth is the

*crusta petrosa*, of which existence he seems to have been ignorant. Salter and Tomes seek their classification from their supposed development, say they are developed from the tegument of the mouth and, therefore, modified skin. We must view the teeth thin as an apparatus peculiar in structure and peculiar, therefore, in function—the structure being adapted to their functions. Therefore, I must reject every classification and regard them as organs '*sui generis.*' Writers tell us that enamel is destitute of vitality; that dentine is not vascular, i. e. has no blood vessels—has no nerves. The sooner we get rid of such views the better. There can be no part of the human economy destitute of vitality. It is a broad statement but nevertheless true. Let an instrument touch the enamel, it is felt and the exact point of contact recognized. Dead matter is foreign to the organism, and nature proceeds to rid the system of it. The vascular and nervous supply to the dentine is as clearly demonstrated by physiological facts as by the scalpel or microscope, nay more so, the eye and the microscope are often deceptive: we may misinterpret a fact but cannot alter it. Salter says dentine is not vascular, i. e. has no blood-vessels, yet he gives us an account of warty hypertrophy of teeth, of necrosis, exostosis, of tumors of various kinds, and finally, narrates two cases of abscess of teeth. Inflamed teeth sensitive to the touch, yet no nerves! inflamed teeth whose dentine on fracture is pink from congestion, yet no blood-vessels! Abscess of teeth yet no blood! causing excruciating pain yet no nerves! The celebrated Joubert has told us that the os uteri is destitute of nerves—he could find none, and it is insensible to the touch. There must have been both vascular and nervous supply to perfect the teeth apparatus; there must be both nervous and vascular supply to maintain this apparatus in its integrity. I am aware that certain pathologists deny that any nervous influence passes along a nerve-fibre which can directly change the rate or character of nutrition of a part; and that some hold that it is an affair of the vaso-motor system.

Whence comes dentine repair, the occlusion of the dentinal tubes, but from vascular and nervous supply? Whence comes the condition of health in which these tubes are kept open?

Finally Mr. Tomes says he can see no reason why the tooth pulp should be supplied so richly with nerves, as no obvious advantage results therefrom; nor can I, if they serve no function of the teeth. No lymphatics have been traced into the teeth—this is a cautious announcement; why not have said there are no lymphatics in the teeth, for the same reason that it is said there are no blood-vessels in the teeth? But we are told that the teeth are composed of inorganic matter. The sooner we get rid of such terms as organic and inorganic the better for progress; it is a legacy left us by chemistry when in its swadling clothes; in its present infantile condition it is still retained, for we yet have in our text-books the division into inorganic and organic chemistry—as if there could be two chemistries; two sets of principles, so that when one failed, the other might be brought into service. I take it, without the presence of the so-called inorganic substance, phosphate of lime, that the human body would be a queer thing; it would, at least, be sans teeth, if not sans every thing. Lime is one of the substances of a large number of organized bodies. It is composed of oxygen and calcium so posited, the one with the other, that as soon as you disturb their relative positions they separate. The organized substance of wheat owes its properties both physical and vital to the positing of the molecules of which it is composed, and polarized light informs us of its structure.

The crystal quartz is as truly organized as the pea which contains the embryo pattern of the plant. The same is true of animal structure, which results from the peculiar arrangement of the molecules of matter of which it is composed. Thus it is as absurd to speak of inorganic phosphate of lime of teeth, as to deny the organization of animal structure. Structure is the result of the action of force upon matter. Function is the result of arrangement of matter by the forces of nature. I repeat that we must regard the teeth as a peculiar apparatus, consisting of many parts curiously correlated for certain purposes. No master of the profession of dentistry, when he examines disease, but takes into consideration the anatomy of the organ, its relation to neighboring organs, and its relation through the nervous system to remote parts of the human organism.

Finally, to show to what lengths classification may be carried, I will mention that the celebrated naturalist, M. de Blainville, pushing analogical induction to its last limits, makes the skin the fundamental organ of the economy; derives all the organs of sense from the skin; produces the apparatus of locomotion from the elasticity of the skin, from which it derives contractility; the apparatus of digestion and respiration a modification of the faculty of absorption. secretion and generation but a modification of the exhalent function. The circulatory apparatus being the only one not immediately derived, he regards this as only an extension of the cellular tissue which is derived from the skin.

### SKIN.

The anatomy, physiology and pathology of the skin is perhaps the most difficult of the human organism. Numerous savans have undertaken the work and arrived at different conclusions in regard to its structure, functions, and pathological conditions resulting from disease. I regard the skin as an apparatus composed of many organs adapted to peculiar purposes. Thus it contains the organs of *tact* in its papillae, into which we trace blood-vessels and nerves, and where the nerves inosculate. The inosculation cannot be traced by the scalpel or microscope, but is a physiological induction. It contains organs of sensation as well as tactile organs which serve to place the brain and nerve centres in relation to the external world. The skin envelopes the entire body, terminating at the external orifices of the body, being continuous with the mucus-membrane which has been erroneously termed an inner skin.

It terminates at the extremities by horny structure called finger and toe nails. The skin contains fibrous tissue arranged into fibres interwoven and crossed, to which it owes its highly important property of elasticity, flexibility and extension, and in virtue of which it protects the subjacent organs, and adopts itself to the varying conditions of the body during life. It contains sebaceous follicles which serve to lubricate and keep soft the parts over which the oily matter spreads. We also find in it hair follicles, glands and sweat-vessels. The skin is highly vascular, containing arteries, veins and capillaries; it is richly supplied with nerves and contains numerous lymphatics. Besides these organs we find a pigment structure which varies in individuals, but is most striking in the different races of men. Thus we have the white, the black, the copper color, the Mongolian color. This membrane is deposited under the epidermis. The color of the skin is interesting to the naturalist; it is *one* of the distinctive characteristics of the Negro, and is in correlation with other peculiarities of that race, which taken together, demonstrate that he is a distinct type of man—genus ape according to the *creation theory*, or a separate divergence from a parent stock or common protoplasm, but so far removed that we cannot trace his consanguinity to the white race. The result has been, on whatever hypothesis you may select a distinct species of animal, it would seem that nature has so marked the different races that we might be warned against the crime of miscegenation. Whom God has separated, let not man join together. The mulatto is a hybrid which dies out in the fifth generation—the albino is still a negro. The great geographer, Malte Brun, tells us of pie-bald negroes. But this is not the occasion to discuss the important question of races in the United States.

One of the most interesting set of organs of the skin apparatus is the lymphatics. The lymphatic vessels originate according to Cruveillier and others in a fine net-work which may be displayed by mercurial injection that passes into the lymphatic vessels. This net-work of lymphatics is situated immediately under the epidermis, and above the layer of capillary blood-vessels. Cruveillier tells us that he has often injected the capillary (reseau) net-work in different regions of the body, and that M. Forman, who has given special attention to this subject, succeeded in showing this net-work and giving a perfect idea of its disposition, which forms when injected with mercury, a silver layer under the epidermis. From this net-work arise the branches which traverse the skin in every direction ; and from the internal face of the dermis lymphatic vessels proceed. The cabinets of the faculty at Paris contain many beautiful preparations representing the lymphatic vessels of the neck and head, prepared under the direction and supervision of Cruveillier, by direct injection of the cutaneous net-work of lymphatics.

### NAILS.

The nails in man, are hard, flexible, elastic bodies which occupy the dorsal face of the last phalanx, and appear to support and protect the skin-pulp, so that the entire digital pulp serves for the *tact.* They present the appearance of scales of horn. The root of the nail is implanted in the fold of the skin which is called its matrix. The body of the nail is free on its upper face, but attached below to a net-work of extreme vascularity which gives it the rose color. The nails terminate in a free border. Nails are regarded as a product of secretion, and like the enamel of the teeth, void of vitality. I cannot admit the presence of dead matter as forming an essential part of the living organism. Nails grow from the root or matrix, and also from the capillary structure adherent to the body of the nail. Nails continually increase in length but not in thickness.

### HAIR.

The hair, too, is regarded as a secretion, and therefore, a lifeless appendage to the living human form. This crowning glory of a woman—a bundle of dead hay ! Lifeless, forsooth, because the anatomist cannot trace blood-vessels and nerves, and because the microscopist cannot see them with his glass eyes. Earth, air, fire and water were regarded for similar reasons as elementary bodies. One hundred years ago no one knew the composition of so universally diffused a substance as water; and combustion was supposed to be due to a principle of *lightness* called phlogiston; when a substance was burnt, the products increased in weight by losing this principle of lightness, phlogiston.

The hair is less devoloped in man than in any terrestrial animals, and hence the necessity of clothing. "Man differs conspicuously from all other primates in being almost naked; but a few short straggling hairs are found over the greater part of the body in the male sex, and fine down on that of the female sex. In individuals belonging to the same race, these hairs are highly variable, not only in abundance but likewise in position; thus the shoulders in some Europeans are quite naked, whilst in others they bear thick tufts of hair. There can be little doubt that the hairs thus scattered over the body, are the rudiments of the uniform hairy coat of the lower animals. The fine, wool-like hair or so-called *lanugo* with which the human fœtus during the sixth month is thickly covered, offers a more curious case. It is first developed during the fifth month on the eyebrows and face, and especially round the mouth where it is much longer than that on the head. A moustache of this kind was observed by Eschricht, on a female fœtus: but this is not so surprising a circumstance as it may at first appear; the two sexes generally resemble each other in all external characters during the early period of growth. The direction and arrangement of the hair on all parts of the fœtal body are the same as in the adult, but are subject to much variability. The whole surface including even the forehead and ears is thus thickly clothed; but it is a significant fact that the palms of the hands and the soles of the feet are naked like the inferior surfaces in all four extremities in most of the lower animals. As this can hardly have been an accidental coincidence, we must consider the woolly covering of the fœtus to be the rudimentary representative of the first permanent coat of hair in those mammals which are born hairy. This representation is much more complete in accordance with the usual law of embryological development, than that afforded by the straggling hairs on the body of the adult."—(Darwin).

Such are the important conclusions of Mr. Darwin from the study of the human hair; he is, however, at a loss to account for the long hair in women, and must try again the effects of his magic wand—sexual selection. The hair presents differences according to sex, age and race. The negro presents much less development than the white race, resembling in texture rather wool than hair. The hair from that extremity attached to the skin is contained in a kind of follicle, which is the formative organ. The follicle is contained in the sub-cutaneous cellular tissue, and is prolonged to the surface of the skin by a sort of membraneous canal. For the rest, the hair is filiform, very flexible, variable in length, diameter and color, and has received different names according to the region of the body occupied by it.

It is evident from the structure of the skin and the numerous organs contained, that its functions must be as varied. It envelopes the body, and by its sensibility places the brain in connection with the external world; it is also in connection with the centres of the nervous system; it contains in the fingers tactile organs, discriminative organs —organs of perception in distinction to organs of sensibility. It contains exhalent organs, and is, therefore, a great depurator of the blood—a vast eliminator of foreign matter—morbific matter. In virtue of its lymphatic system it is a mighty absorbent. The importance of the skin in treatment of disease is thus apparent. It is an apparatus which is too little considered in reference to disease by the professor of medicine.

### SYPHILIS.

No branch of pathology has given rise to more discussion in regard to its origin than syphilis. The factions are divided into those who contend that syphilis existed in ancient times, and those who date it from the latter part of the fifteenth century, and ascribe its origin in Europe to Columbus' sailors. I do not hesitate to give my opinion that syphilis is a disease rather of civilized than savage life. The most ancient account of this disease is to be found in a Hindo work, by Sucrutas, who speaks at length of various ulcerations on the genital organs, and eruptions on the palms of the hands and the soles of the feet.

A work published in 1863—La Medecine chez les Chinois, par le Capitaine Dubry leaves no doubt that venereal and syphilitic diseases were observed and described in China in the most ancient times. "It is there stated that an author by name of Hoan-ty, who lived 2637 years before Jesus Christ, wrote on gonorrhoea with its complications of cystitis, nephretis, and epididymitis. He also is said to have described chancres, of which he notices two kinds, one of which suppurates freely, the other emits only a serous matter; he notices also the accompaning tumors." The author goes on to describe the disease in the most unmistakable manner. Hippocrates speaks of ulcerations of genital organs, tumors of the groin, carnosities of different portions of the body, extensive pustular eruptions. Celsus describes two varieties—ulcera sicca, ulcera humida: "Bumstead."

The lamentations of King David over the sharp pains in his bones, is evident of Holy Writ on the subject; and if Job was not afflicted with syphilis, then the devil forgot the greatest infliction he could have imposed, which is not likely from a biblical stand-point of view.

Ricord believes that the epidemic of the 15th century was glanders, not syphilis. He states that an analogous idea has been suggested by Van Helmont, who derives syphilis from farcy in consequence of bestial connections. Cazenaue believes that the epidemic of the 15th century was typhus.

Professor R. B. Winder has shed much light in regard to its American origin, in his excellent and instructive paper concerning the teeth of Indians, a large collection of which he had an opportunity of examining in the Anatomical Museum at Washington City. In the hundreds of skulls of Indians of all tribes and natives of North America, the teeth, with a few exceptions are perfect—not a blemish. The exceptions proved to be in the half-breeds. He also mentions the instructive and important fact that syphilis introduced by sailors into one of the islands of the Pacific, destroyed nearly all of the inhabitants. What then are we to make of the fact that Columbus makes no mention of so fatal and terrible a scourge among the Indians? I have not been able to get an account of the teeth of the Indians of Central America and the West Indies. It would throw much light upon this question, in view of the recognized effects of syphilis upon the teeth of those to whom it had been transmitted by heredity.

In considering the effects of diseases of the human organism upon the teeth, we must take into consideration the effects of climate, and also the differences in structure of these organs in races of men on the earth. Races of men, like other fauna, flourish best in particular zones. When transferred from one to another these changes are marked. It has been said the white races at present inhabiting North America are exotics, and without constant accessions from Europe, would degenerate and finally become extinct like the pre-historic race whose remains are to be found in the mounds scattered throughout the West and South. It has been asserted that this deterioration is exhibited in the teeth of the American people; that their teeth are inferior in quality to the European. Whatever may be said of the *white* race, it is unquestionably true of the "*Negro.*" The splendid display of ivory of a century ago by the negro, no longer obtains. Formerly the fine teeth of the negro was proverbial, whose dazzling whiteness was increased by the contrast with his burnished envelope of ebony. A single African skull in some collection I have read of, presents perfect teeth. At this period the teeth of the negro are as proverbially indifferent as formerly they were excellent. I do not ascribe this degeneration to the kind of food. The negroes of the South were well cared for in every particular ; their food was plain but of the most nutritious kind. Sufficient date have not been collected to enable me to do more than suggest that such conditions of deterioration are worthy of consideration in discussing the effects of disease upon the teeth. One benefit I think will ensue from such discussions even by those so imperfectly informed as myself, which is, that the attention of the profession will be directed to the effects of various diseases upon the teeth and organs wanting in vitality.

We are indebted to Mr. Hutchinson, of London, for calling attention to the alteration of the teeth caused by syphilis. This condition he states is confined to hereditary syphilis, and is only exhibited by the permanent teeth. In diagnosing inherited syphilis he says, "We have very valuable aid furnished by the *shape* of the incisor teeth. In these patients it is very common to find all the incisor teeth dwarfed and malformed. Sometimes the canine are affected also. These teeth are narrow, rounded and peg-shaped: their edges are jagged and notched ; owing to their smallness their sides do not touch and interspaces are left. It is, however, the *upper central incisors* which are the most reliable for the purposes of diagnosis. When the other teeth are affected these very rarely escape, and very often they are malformed when all the others are of fairly good shape. The characteristic malformation of the upper central incisors consists in a dwarfing of the tooth, which is usually both narrow and short, and in the atrophy of the middle lobe. This atrophy leaves a single broad notch (verticle) in the edge of the tooth and sometimes from this notch a shallow furrow passes upwards on both anterior and posterior surface nearly to the gum. This notching is usually symetrical ; it may vary much in *degree* in different cases, sometimes the teeth diverge and at others they slant towards each other." He then refers to a wood-cut to illustrate the deformity, and proceeds, "I have never seen such teeth excepting in patients of this class. In the majority of cases, however, the condition of the teeth is sufficient only to excite suspicion and not to decide the question. In a few rare cases, only one of the upper incisors is malformed, the other being of natural shape and size. In addition to the peculiar malformation above described, there are others, which, although less characteristic, are yet very valuable to a trained observer. It is only in the permanent set that any peculiarities are observed; the first set are liable to premature decay but are not malformed "

I am indebted to the kindness of Dr. I. L. Atkinson, of Baltimore, for the privilege of exhibiting two casts of the upper teeth, one of which corresponds wonderfully with the description of syphilitic teeth given by Mr. Hutchinson; in the other the teeth are deformed and notched. They were both taken by Dr. Atkinson from patients the subject of hereditary syphilis. Dr. Atkinson related to me a circumstance of interest in discussing this subject.

At a meeting of medical gentlemen when this question was discussed, a physician arose and announced that one of his children had notched teeth which supervened from a severe and protracted illness of scarlet fever. He was a man in robust health, and averred that he had never been the subject of syphilis, nor could he trace on either branch for the child any such taint. The child remained long after its attack of scarlet fever in poor health, but regained both health and strength, is hearty and robust and has never given the slightest symptom of syphilitic taint. Mr. Slater's Dental Pathology and Surgery, says of syphilitic teeth, "These conditions are usually, though not *always* confined to permanent teeth, which Mr. Hutchinson attributes to the fact that hereditary syphilis does not develope itself during intra-uterine life. Mr. Hutchinson supposes that attacks of syphilitic stomatitis are necessary to produce these malformed teeth, but he says, I quite agree with Mr. Tomes that such an idea is unnecessary as an explanation of the condition in question, and it seems to me that the malformation of the teeth is probably more the result of perverted nutrition than of inflamation."

A most interesting discussion of the manifestation of syphilis in the teeth by the Association of Surgeons practising Dental Surgery, is reported in the London Lancet May, 1876. From this discussion it will be seen that opinion in regard to this lesion as diagnostic, is by no means settled. Mr. Napier read a paper on the subject. He hesitated to admit the manifestation of a semi-lunar notch upon the two front teeth as an infallable indication of syphilitic taint. He called attention to the rarity of its appearance in comparison with the number of certified inheritors of syphilitic disease. He attached importance to the fact that it is wholly confined to teeth of second dentition, whilst syphilis asserts itself from the commencement or very early in infantile life. Dr. Drysdale expressed his surprise that Mr. Napier with his great opportunities could express a doubt as to the character of syphilitic teeth. Mr. Mason agreed with Mr. Napier. Mr. Coleman said they were almost invariably connected with syphilis. Mr. Risdon being connected with the Children's Hospital, doubted whether the teeth spoken of as syphilitic were really so; he thought they might be the result of struma, and as they appeared after scarlet fever they might arise from mercury. Mr. Jonathan Hutchinson did not deny that disbelief in his theory was very prevalent in all ranks of the profession save in that which is concerned with diseases of the eye, and he did not wonder at it, nay, rather admired the scepticism of those who thought that the malformation of a single pair of teeth could not be indicative of specific disease. Had it not been for his experience of diseases of the eye, he might have joined their ranks. He was surprised to find at the Pathological Society, sometime since, how many did not understand the peculiar types of syphilitic teeth. If he saw a pair of central incisors with the peculiar lunal notch, he would feel certain that the possessor of them had been syphilitic. Other teeth no doubt were often malformed, but not in that peculiar way, and he should, therefore, draw attention to those teeth only, viz: the *permanent central incisors of the upper jaw.*

He thought that the reason why other teeth were so affected, was probably owing to the fact that those suffering from syphilis had taken mercury, which also left its mark upon the teeth, and he inferred that those who had taken least mercury would present the most perfect syphilitic teeth. An interesting case had come under his observation.

Mr. Waren Tay was seeing his patients at the Skin Hospital, when a woman with acne on her face sought his advice. He found the peculiar syphilitic teeth and no other indication of that disease. She was sent to me for examination. There were no syphilitic symptoms, for she stated that her sight was perfect, but on examining her I found that she was suffering from defective vision, and had marked choroiditis, and this latter, in conjunction with the malformed teeth, is an almost certain proof of inherited syphilis. Concerning mercurial teeth, he thought they were stomatitic, and his attention had been drawn in that direction, because they were nearly always found in connection with lamellar cataract. Lamellar cataract is always found in connection with ill-formed teeth, and were generally connected with convulsions in infancy. He thought that the convulsions caused lamellar cataract; the mercury given to cure these caused the malformation of the teeth, and just as the central upper incisors were test teeth for syphilis, so the *first* molars were the test teeth for mercury; the other teeth not being so affected was owing to the fact that they were developed at a period when stomatitis was not so frequent.

Mr. Hutchinson finally suggested that Fellows of the Association should attempt to discover whether any peculiar signs were to be found in the teeth of those of a rickety or scrofulous diathesis, for information on those points was of the most vague description, and that in opthalmic practice was to be found the best confirmation of syphilitic teeth. There were present the most distinguished members of the profession.

For my own part, when I reflect that syphilis is a disease which may affect every organ, apparatus, system and tissue of the economy, I cannot doubt that it affects the teeth. There is no tissue which may not be assailed by the dread disease. The nervous systems, the glandular systems, the skin, the mucous-membrane, the internal organs concerned in digestion, assimilation, elimination, and nutrition. But no matter what organ or system may be assailed, there are peculiarities of manifestation by which the experienced practitioner may detect it. Multifarious as are its forms when it invades the skin, we find the syphilitic mark, the syphilitic lesion. When the hair is affected there is the syphilitic sign; when it affects the mucous-membrane and fauces, it leaves its mark, on the liver it leaves its peculiar impress. Syphilitic headache differs from migraine. Syphilis is a specific disease, and is specific and peculiar in its manifestations. Now as the teeth form a most important apparatus of the human organism, developed and grown like other tissues under the operation of the forces which control nutrition; as they are supplied with blood-vessels and nerves like other organs, I do not see why they should not be affected by those diseases and those agents which affect the general economy. Mercury affects the teeth; general ill-health causes deterioration of the teeth; scarlet fever we have seen affects the teeth, and finally we have evidence that syphilis affects the teeth. The question remains, how? Does syphilis leave its diagnostic mark? I have no doubt of it. It is a question of the action of force on matter. Syphilis makes its peculiar mark on the skin, on the mucous-membrane, as far as we can infer on the nerves, the nervous manifestations are at least peculiar; on the liver, and why not on the teeth? If it does not, the teeth are exceptions to the rule. I believe that it does when it attacks these organs, and the thanks of the profession are due to Jonathan Hutchinson for having pointed out the "*tooth test*" *for hereditary syphilis.*

### SKIN, NAILS AND HAIR.

In regard to syphilitic affections of the skin, nails, and hair, I shall have little to say. The subject is too extensive to be treated at length within the limits of this paper.

From what has been said of the complex organization of the skin, and the many important functions performed by the organs which compose it, we would not expect it to escape the ravages of a disease of such duration and persistence as syphilis—of a disease which confessedly affects the blood, whose integrity is necessary to the healthy function of every part of the animal economy. Accordingly we find syphilis manifested by disease of the hair follicles, and likewise of the nails in their matrix and body, giving rise to the various diseases to which these organs are liable. Dermatologists have classified diseases of the skin according to the anatomical lesion presented to the eye of the observer. Thus we have *exanthemata*, which includes a large class of dermoid affections such as erysipelas, roseola, scarlatina, urticaria— *Vesicula*, as varicella, eczema, herpes, scabies, characterized by an eruption of vesicles more or less numerous—Bullæ, as pemphigus, rubia—*Pustula*, as variola, vaccine, glanders, eczema, impetigo, acne, porrigo — *Papulas*, as lichen, prurigo—*Squamous*, as psoriasis— *Tubercula*, as eliphantiasis—*Macula*, including the various discolorations of the skin. Besides, there are numerous diseases which cannot be classified under any of the orders enumerated, such as lupus and pellagra. There is scarcely one of the diseases mentioned above which syphilis may not give rise to, and in many of them special and local treatment will be found necessary, in addition to the specific remedies generally used in the treatment of syphilis. But I would call attention again to the important fact, that no matter what variety of skin disease may be manifested, syphilis leaves its mark; as in the teeth, we have test teeth, so when the skin is affected, syphilis impresses its test mark. These are not always capable of being described so that they may be recognized, but to the experienced practitioner they are evident. Experience educates the eye as it does the touch. Indeed there is but one sense, that of "touch." When the waves of luminous ether "touch" the optic nerve, vision is the result: the motion is conveyed to the brain and translated into vision. The optic nerve is as much a tactile organ as the papillae or nervous pulp of the fingers. When the waves of sound "touch" the auditory nerve, it is recognized by the brain. When odoriferous bodies "touch" the olfactory apparatus, odors arise. When sapid bodies touch the gustatory apparatus, taste appears. In fact we feel with the brain, see with the brain, smell, taste and hear with the brain, in consequence of impressions upon our nerves, called nerves of special sense, which are carriers only, and all a modification of the touch.

It is then the "tactus eruditus" which so often enables the practitioner to recognize disease, when he fails to make others comprehend the steps by which he arrived at his conclusions.

Syphilis should be classed with the so-called zymotic diseases. The smallest quantity of virus serves to contaminate the entire organism. It is a self-limiting disease like small-pox, and like small-pox is often capable of elimination by the forces of nature; it may be modified in its course by remedies—may be cured by remedies—may prove fatal in spite of remedies. It has its period of incubation; its eruptive stage; its period of progress. An attack of syphilis gives partial or complete immunity from a second invasion. It may be transmitted by heredity, and in the opinion of some, the immunity gained by the parent may be transmitted to the offspring.

The second stage of syphilis, as it is called, is characterized by affections of the skin, more often of the exanthemata, whose duration is variable. But what is of great diagnostic value, is that the eruptions of this stage are symmetrical—affect the two halves of the body at the same time. We find symmetrical ulcers in the tonsils, not spreading either in width or depth. There is also loss of hair. The exanthem usually takes from a fort night to a month before it is fully out, and about two months to decline. The color of the eruption is of a peculiar coppery hue, which is one of the syphilitic "test marks." The rash may be merely congestive, it may be squamous, papular, postular, bulbous, eczematous. As a rule, the eruption of the secondary stage involves only the superficial layers of the skin, differing in this respect from the tertiary manifestations. Although there are a variety of eruptions, it does not imply a difference in the nature of the virus—this depends upon the tissue attacked. Peculiarities of constitution may determine the kind of eruption—the same kind of chancre in different individuals may be followed by different kinds of eruptions. The affections of the skin in the tertiary stage are not symmetrical; they involve ulceration of greater or less depth, and consequently leave cicatrices. According to Cazenane, syphilitic cicatrices present a peculiar aspect—they are round, depressed, of a dull white color, furrowed sometimes with deep bands.

Syphilitic eruptions present a coppery hue; they never present the red color of inflamation—this is one of the syphilitic "test marks." It would require a volume to treat of syphilitic eruptions of the skin, it would require a disquisition on dermatology to do justice to the subject. I shall, therefore, on this occasion, hold myself excused for not dwelling at greater length upon the lesions caused by syphilis, contenting myself with having pointed out some of the syphilitic skin marks arising in the progress of the disease. I feel assured that you will readily excuse me, and probably thank me for my brevity in this respect.

Syphilis is a disease which has been much studied of late, and much progress has been made towards unravelling its nature, the laws of its progress, its termination, as well as its modes of transmission. Yet much remains to be done; it cannot be said that the disease is understood in its length and breadth. Investigators differ among themselves in many important particulars. Thus, Hutchinson rejects the "duality theory" of syphilis as an unnecessary hypothesis, whilst Diday says secondary attacks are not so rare as has been supposed. Again, Hutchinson holds that what we call the tertiary stage is only the sequel of syphilis; is never spontaneously cured, and liable to remarkable relapses when relieved, the lesions being due to the alteration in the constitution of the economy, and not the result of the continuous action of the virus.

In conclusion, I trust that you will bear in mind the request made by Mr. Hutchinson to the association of surgeons practising dentistry, and endeavor to ascertain whether any peculiarities are to be found in the teeth of those of a rickety or scrofulous diathesis. And I would ask you to give the results of your observations of the effects of all diseases, which in the course of a long and active practice, may be submitted to your inspection. We must look to your special branch of Surgery for information on all such points. The appeal I am sure will not be in vain, when I remember the rapid strides you have made within a few years. You have lifted your profession from being merely a mechanical calling, to be a branch of Surgery; an art based like other specialties upon the broad principles of Anatomy, physiology and chemistry. You will not deem me intrusive, I am sure, if I caution you against relying for your opinions upon "authority." This is the bravest progress of medicine. I dislike to be knocked down by "authority." Virchow says so, Neimeyer thinks so, and the like, which is supposed to be quite a sufficient answer to any views of your own. I have a high regard for worth and excellence. I honor the great men of our profession, but at the same time it will do no harm to do a little thinking for ourselves. I fear that I am not an "idol" worshiper, and sometimes wish that an iconoclast would make his appearance. In the history of the world they have been instruments of progress, of intellectual and moral development. Jesus Christ was a great iconoclast, although many an idol has been erected in his name. Gallileo in his time was a great iconoclast, although he perished, for they would not permit the old man to *see* his friends until he was *blind.*

Lord Bacon was a great breaker of idols—he broke the idols of the tribe, the idols of the den, the idols of the market, the idols of the theatre, as well as the worship of the ancient philosophy. Such was he at least for English speaking people, although Draper tells us he knew nothing of philosophy. Thus is Draper an iconoclast. Darwin, and Huxley, and Tyndall, and Spencer, are all iconoclasts

Robespere and Oliver Cromwell were iconoclasts of the first water. And movement, and progress, and intellectual and moral development has been the result. Morality is based upon intellectual progress, and the time may yet come when a man will do *right* from a principle, and not because of reward in heaven, or fear of damnation in hell.

When I am met with " authority " and knocked on the head by it—stunned—can't answer a word, I sometimes think of a letter which Oliver Cromwell wrote to the Scotch Presbyterians in reply to theirs, urging certain dogmas to be imposed upon the nation. He concludes thus, " I beseech you brethren, in the bowels of Christ, to think that it is *possible* that you may be mistaken."

The paper was discussed by Drs. W. H. Atkinson,* and J. J. Caldwell.

Dr. Caldwell.—I want to say that my working brother, Dr. Scott, will not be here to morrow. I merely wish to state I respect a man who is dogmatic. If he is true, I want him to live by what he says, and die by it.

On motion of Dr. Foster, the Association at fifteen minutes of two took a recess until half-past two.

*Note.—The reporter, being an amateur, hopes he may be excused for not getting Dr. Atkinson's remarks; even fully enough to make a fair report.

AFTER RECESS.

Dr. Foster.—I should like to hear from Dr. Scott on this subject. While I think the paper was exceedingly able, I think the Doctor has not entirely exhausted the subject.

Dr. Scott.—I have nothing to say, except that I should like to hear from the gentlemen present, especially on the syphilitic taint, and I should like to hear a discussion upon any point I made with regard to the teeth being dermoid tissue. I cannot classify them at all. I should like to hear from the gentlemen present.

Dr. Foster.—Just in regard to that one point. How are we to find out if cause of certain marks upon the teeth is syphilis. The question arises whether we can ask the patient about his father? Whether he has been a fast man or not? Whether he was known to have had syphilis or not is the next question, and one that is not asked. That comes again into the hands of their medical adviser.

To be sure, Mr. Hutchinson has had a great deal of experience, but whether he can say these things positively I very much doubt. They argue on these things and present them to the Dental profession; the Dental profession accept them as facts without judgement, that they are syphilitic. Why are they syphilitic teeth? Why will not other things manifest themselves in malformation of the teeth. I believe that many times when they say these teeth are syphilitic they are mistaken.

Prof. Winder.—I do not know of any light I can shed on this subject. It is one I have not investigated. That these teeth may be manifestations of syphilis may be true; that it has been demonstrated to be so, I am inclined to doubt.

It is simply a theory. I do not know that Mr. Hutchinson himself is as positive as he might be on the subject. It is a suggestive question. One that puts men to thinking whether it is from the action of diseases or other surroundings. I cannot certainly let myself down to believe these teeth to be a certain diagnosis of hereditary syphilis; still, at the same time, it may be so. It is a question that must be circulated. There must be a great deal of statistics taken to establish it as a fact. We know positively, that any interference with the development of the teeth at any time during their growth, will interfere with them precisely as it would with any other organ. I believe with Dr. Scott, that teeth are governed by the same physiological laws that other organs are.

These causes, of course, would interfere, but as to the specific action of syphilis, I do not suppose anybody claims to understand or know anything about. That it is hereditary, we have the best knowledge in the world. Therefore, I would not like to say that it could not leave its manifestations upon the teeth.

Dr. Caldwell.—I think, Mr. President, that Dr. Hutchinson claimed to have concomitant testimony to prove his theories. Syphilis manifests itself at one time in one portion of the body, and then in another.

Dr. Atkinson.—A word as to whether these teeth are dermoid structure. Whether they are dermal or hypodermal or epidermal?—They are all. They are epidermal in the enamel, dermal in the dentine, and they are hypodermal in the cementum and pulp.

Dr. Foster.—If there are no other gentlemen wishing to speak upon this paper, I move you that it be passed until to-morrow, when other papers will be read on this subject. The motion was carried.

Dr. Winder.—I move that we suspend the rules, as I have something I wish to bring before the Society. Carried.

Dr. Winder.—There are two things I wish to speak about, one of which Dr. Waters mentioned in his address of welcome this morning. He states, and I believe it to be a foregone conclusion, that next year there will be inaugurated a great National Convention in New York. I believe that the membership in that organization will consist of the memberships in State Societies, therefore, every man who is a member of a State Society, par excellence, is a member of that organization.

It will be any man's privilege to go there and take part. I think that right on the eve of this thing, it is our duty to see that the requirements of the ethics made by this Society are faithfully copied. Gentlemen who do not like to be bound by ethical laws should not come in here, and subscribe to the ethics. Having promised to practice the profession according to the ethics, he should be compelled to do so.

Every man we place in this Society, as soon as he is a member of it, is entitled to representation in that National organization. No man can be a member of the National without being a member of a State Society. That is the matter I wished to speak of in regard to Dr. Waters' paper.

In regard to what fell from the President's lips this morning, I wish to call attention to that part in which he alluded to the printing of the proceedings. I do not know the financial condition of this Association. I know that as one member of it I am willing to be taxed, and I do not want the Society to pass a year without printing the proceedings. I want some action taken to provide a means for this printing. I propose that when there is a full meeting, the Treasurer make a report so that we can take action. This is a matter of importance to us; we are visited by gentlemen. We have had to-day a paper from one of them that was very interesting, and I think we should weigh the matter well before we decline to print our proceedings.

Dr. Foster.—I agree with Dr. Winder in regard to the publication of the proceedings, and I think it well enough to bring it up to morrow morning. I do now make it a motion that the Treasurer be requested to make a report of the state of the treasury. I think none of the gentlemen present would object to a slight taxation. The motion was carried.

The President.—Was on the Publication Committee for three years, and I know we have had a great deal of trouble in getting printers and then paying them. This year we had nothing whatever to pay the printers. It kept growing worse and worse, and I thought it well to cut down expenses a little.

On motion of Dr. Winder, the Association adjourned.

---

*American Academy of Dental Science.*

